# Chitosan: A Versatile Biomaterial Revolutionizing Endodontic Therapy

**DOI:** 10.7759/cureus.62506

**Published:** 2024-06-17

**Authors:** Akash Thakare, Shweta Sedani, Simran Kriplani, Aditya Patel, Utkarsh Umre

**Affiliations:** 1 Conservative Dentistry and Endodontics, Sharad Pawar Dental College and Hospital, Datta Meghe Institute of Higher Education & Research, Wardha, IND

**Keywords:** bio-compatibility, root canal irrigants, root canal bacteria elimination, chitosan nanoparticle, polymeric nanoparticles

## Abstract

Owing to their nanoscale dimensions, nanomaterials have special chemical and physical properties that set them apart from their bulk counterparts. The exterior dimensions of a minimum of half of the particles span several nanometers in their size distribution. Silver nanoparticles (AgNPs) are one type of nanomaterial that has been widely used because of their strong antibacterial properties, which can kill bacteria that are resistant to many drugs. Due to its potential for regulated release, localized retention, and safeguarding the active ingredients against environmental or enzymatic deterioration, nanoparticle technology has also emerged as a promising medication delivery method. The techniques for creating nanoparticles can be easily scaled up and used for a wide variety of medications. Since polymeric nanoparticles are biodegradable, biocompatible, and have more readily available formulation techniques than other nanoparticle drug delivery approaches, their range of applications has been expanding. Chitosan, also known as deacetylated polysaccharide, is a straight-chain cationic polymer that is typically a cationic copolymer. It can be generated naturally or by deacetylating chitin. Consequently, it contains an extensive array of biomedical applications, such as efficient healing of wounds, regeneration of tissues, regeneration of bone, and anti-infection. Because of its functional diversity, accessibility, and being both biodegradable and biocompatible, it has a wide spectrum of uses in dentistry. Recent research on chitosan-based nanoparticles is founded on the field’s growing comprehension of the characteristics of chitosan and techniques for chemical or physical modification that are used to optimize the drug loading and release characteristics of the nanoparticles.

## Introduction and background

Dental problems have been identified as one of the significant contributors to the substantial hazard to human health [1]. Dental pathologies are multifactorial, encompassing the oral microflora, the genetic makeup of the host, the standard of living, the oral microenvironment, and so on [2]. Among the prevalent illnesses are endodontic diseases, which arise from infections caused by opportunistic microorganisms and active inflammatory reactions within the dental pulp [3]. As a result, endodontic research has garnered interest from the scientific community, primarily focusing on both the preventive and therapeutic elements. The major objective of endodontic therapy is to eliminate the microbes and injured tissues present in the intricate three-dimensional (3D) endodontic system [4]. The stage of cleaning and shaping, which involves the removal of the biofilm of bacterial origin arising from the anatomic and instrumented parts of the root canal system, constitutes a vital stage in the process [5]. In the most complex endodontic anatomy, an active 3D method is used to target the infected pulp tissue and microorganisms by allowing the irrigants to reach deeper locations and ensure effective disinfection [6]. To prevent the risk of reinfection, the primary objective of these therapies should be the removal of bacterial colonies and associated biofilms [7]. Furthermore, owing to their varied hydrophobic nature, hydrophilic properties, and molecular sizes, the antibacterial medications that are currently available face challenges entering the dense extracellular matrix [1]. This barrier matrix, which is composed of tightly packed molecules, creates a significant obstacle, making it difficult for the medications to breach the matrix and target the bacteria effectively [8]. As a result, these antibacterial therapies frequently have decreased potency, making it difficult to completely eradicate the infections [6]. Even after a thorough cleaning and shaping of the root canal, residual bacteria may still persist within the tubules protected by the matrix [9]. Hence, to improve the outcomes of endodontic treatments, the challenge lies in developing antibacterial drugs capable of overcoming these obstacles and providing deeper penetration with thorough elimination of the microbes [4].

## Review

Search methodology

To obtain pertinent and thorough information for this review article, a rigorous literature search was carried out, utilizing a range of search terms. The main search phrases were “chitosan,” “biomaterials,” “endodontic therapy,” “antimicrobial properties of chitosan,” “chitosan in dental applications,” “biocompatibility of chitosan,” “chitosan nanoparticles,” “chitosan-based endodontic sealers,” and “chitosan in root canal treatment.” To find studies, reviews, and clinical trials that examine various functions of chitosan in endodontics, its efficacy in enhancing treatment outcomes, and its potential to improve the antimicrobial and regenerative properties of endodontic materials, these terms were used across a variety of scientific databases, including PubMed, Scopus, and Google Scholar. A total of 274 records were identified through database searching. After duplicate removal, 223 were excluded, and 51 final full-text articles were included.

**Figure 1:**
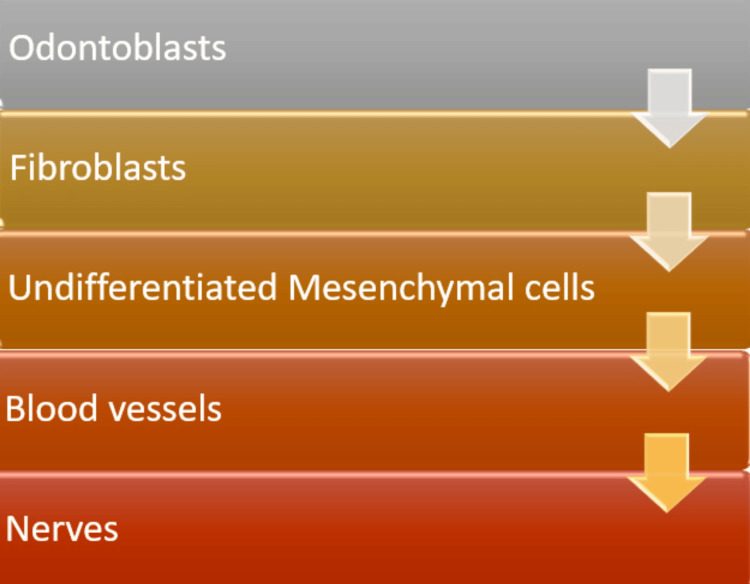
Various components of the pulp tissue Image credit: Akash Thakare

The major hindrance to root canal disinfection and the potential cause of the recurrence of the infection are potent microbes that are present in the dentinal tubules. All of the root canal walls are covered with a smear layer that is 1-2 µm thick after the chemo-mechanical preparation [10]. The remnants of necrotic pulp tissues, along with the biofilm, which consists of remnant bacteria, organic materials, pieces of odontoblastic processes, and inorganic dentin debris, are protected in this smear layer. Various components of the pulp tissue are presented in Figure 1 [11].

The smear layer may prevent intracanal medication and the root canal irrigating solutions from penetrating into the dentinal tubules, which could weaken the binding of the filling materials with the canal walls [12]. Rotary files are not enough to remove the microbes from the complex root canal space. Irrigating solutions are essential for clearing the debris. These solutions act as lubricants and help in the breakdown of both inorganic and organic material while having a strong antibacterial effect during the instrumentation stage [13]. Due to the exceptional antibacterial qualities of sodium hypochlorite (NaOCl), it is highly preferred among irrigating solutions. By producing hypochlorous acid and releasing chlorine, which is a very potent bactericide, NaOCl functions as an antiseptic. Furthermore, the breakdown of the necrotic pulp tissue by free chlorine from the NaOCl occurs as a result of the dissolution of the amino acids in proteins [14]. The presence of microbes leads to the failure of the root canal treatment. Due to their ability to endure harsh conditions, bacteria are able to enter dentinal tubules along the canal walls and form biofilms. These bacteria are more resistant to medications, chemotherapeutic agents, and sealers [10,12]. The presence of bacteria after the therapy, prior to obturation, is closely related to the effectiveness of root canal therapy over the long and short terms [15]. Therefore, it is imperative to improve the efficacy of the disinfection methods and solutions in endodontic therapy. Since there is limited success of these medications or root canal irrigants to entirely remove infections, other approaches will be required to get around this limitation [16]. By creating effective antibacterial nanoparticles, nanotechnologies have recently become a cutting-edge therapy option for tooth infections. Due to their exceptional properties, nanoparticles, which can be produced chemically or biologically, are being utilized more in the pharmaceutical and biomedical industries [17]. Nanomaterials are defined by the Council of the European Commission as natural, synthetic, or supplementary materials that contain particles that can be unbound, mixed, or aggregated. One or more of the exterior dimensions of a minimum of half of the particles in the distribution of sizes fall between 1 and 100 nm [18]. Silver nanoparticles (AgNPs) are one of the nanomaterials that have been widely used owing to their strong antibacterial properties, which can eliminate bacteria that are resistant to other medications. Due to its properties of regulated release, localized retention, and safeguarding the active ingredients against environmental and enzymatic deterioration, nanoparticle technology has also emerged as a promising medication delivery method [15]. With the advancement of technology, the techniques and methods for creating nanoparticles can be easily scaled up and used for a wide range of medications. Since polymeric nanoparticles are biodegradable and biocompatible and have more readily available formulation techniques than other nanoparticle drug delivery approaches, the range of applications has been expanding [19]. Due to its functional diversity and availability, chitosan, which is a naturally occurring polysaccharide with both biodegradable and biocompatible properties, has a wide spectrum of uses in dentistry. Recent research on chitosan-based nanoparticles is founded on the field’s growing comprehension of the characteristics of chitosan and the techniques used for its chemical and physical modification, which can optimize the drug loading and release characteristics of the nanoparticles [18].

Chitosan

**Figure 2:**
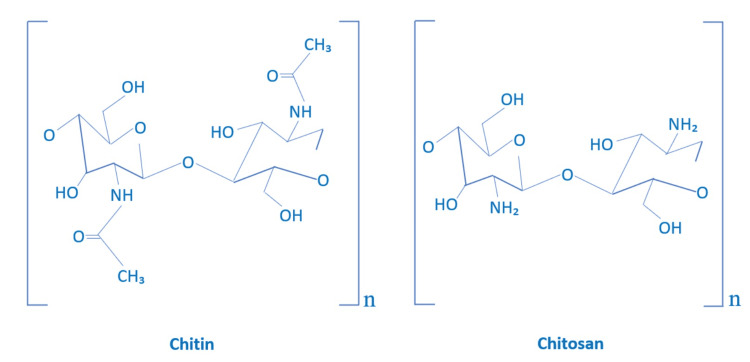
Comparison of chitin and chitosan Image credit: Akash Thakare

Chitosan, which is a natural polysaccharide, demonstrates properties like biodegradability and biocompatibility. It has a multiple spectrum of applications in dentistry due to its accessibility and dynamic functional properties [20]. Recently, it was found that chitosan and its by-products can be ingrained in materials like tissue regeneration, antimicrobial agents, barrier membranes, and dental adhesives to better manage oral pathologies. Moreover, to encourage future studies in the field of endodontics pertaining to chitosan that are more sophisticated, the addition of chitosan compounds to the alteration in order to enhance and modify dental materials has to be thoroughly studied [21]. Chitosan, also known as deacetylated polysaccharide, is a straight-chain cationic polymer that is typically a cationic copolymer made up of 60-100% 2-amino-2-deoxyglucose and 0-50% 2-acetylamino-2-deoxyglucose-D-glucoside. It can be generated naturally or by deacetylating chitin [22]. Primarily present in the exoskeletons of crustaceans, chitin can also be found in certain fungi. Rouget discovered chitosan in 1859 by treating chitin with a hot potassium hydroxide (KOH) solution. This discovery also established the framework for contemporary chitosan manufacturing. Compared to chitin, chitosan has a higher acetylated amino group (Figure 2) [20,23].

The adsorption of metal cations is caused by the nitrogen-free electron pair on the amino group. The percentage of amino groups that can interact with the metal is determined by the average degree of deacetylation [22]. Chitosan is far more soluble than chitin and can be mixed in a diluted acidic medium to create polymers of cationic origin. These polymers can then be mixed with different natural or artificial anionic species, like proteins, lipids, deoxyribonucleic acid, or synthetic polymers that are negatively charged, like polyacrylic acid [24]. Anionic attraction through electrostatics is also caused by the amino acid protonation in acidic solutions. Chitosan has strong biodegradability because lysozyme or chitinase may biodegrade it to a nontoxic residue and because it hydrolyzes glucosamine-glucosamine, glucosamine-N-acetyl-glucosamine, and N-acetyl-glucosamine linkages [25]. Second, by maximizing the quantity of charges with positive origin, acetylation improves biocompatibility by enhancing the contact between chitosan and cells. It has even been shown to have anti-inflammatory qualities and no antigenic reaction. Another important characteristic of chitosan is its hemostatic action, which is mostly because it has the capacity to stimulate endogenous blood coagulation and induce platelet adhesion and aggregation [26]. Through the adsorption of plasma and the coagulation of red blood cells, chitosan can also regulate bleeding. Due to its various processing types, like a solution, film, gel, microparticles, paste, tablet, and microspheres, among others, the properties of chitosan include biodegradability, biocompatibility, hydrophilic nature, antimicrobial action, and biologic activity [22,27]. Consequently, it contains an extensive array of biomedical applications such as efficient healing of wounds, regeneration of tissues, regeneration of bone, and anti-infection. This natural biopolymer is not only affordable because it is made of natural and renewable resources, but it is also among the cost-effective options among various other materials that are available in the present scenario [28]. Making biomaterials from chitosan involves the following methods [29].

Ionic Gelation

Ionic gelation is typically used to manufacture chitosan nanoparticles (CNP). The aqueous acetic acid solution containing chitosan particles and then adding the right amount of sodium hydroxide (NaOH) to the solution to bring its pH down [30]. Next, to create CNP and tripolyphosphate (TPP), an ionic cross-linker is added to the aqueous phases of chitosan and mixed at room temperature using a magnet stirrer. In order to get rid of any contaminants, rinsing of with pure water of the nanoparticles is done. The concentration of chitosan, TPP, temperature, pH, and reaction duration can all have an impact on the physical and chemical properties of the particle, as was previously described [31]. Vaezifar et al. [32] discovered that 1 mg/ml of chitosan, 1 mg/ml of TPP, and 60 minutes of reaction time are the ideal values. After more investigation, it was discovered that the CNP exhibited a zeta potential of around +31 mV, an average size of approximately 90-100 nm, and a polydispersity index of about 0.22 under ideal circumstances. Furthermore, the CNP produced using this technique are extensively employed as gene and enzyme carrier systems and drugs and are crucial for both in vitro and in vivo processes [33]. Many scientists are currently working on improving the way that medications or genes bind to nanoparticles of chitosan. Tang et al. [34] discovered that the following are the ideal circumstances for enzyme immobilization: for approximately 15 minutes at 40 °C, 1 mg of neutral proteinase was immobilized on CNP. The yield of enzyme activity under optimal circumstances was 84.3%. Furthermore, Elzatahry and Mohy Eldin [35] discovered that the superior mucoadhesive qualities of metronidazole in CNP make them suitable for colon-specific administration. In the meantime, new research revealed that the medication is gradually and carefully delivered from the optimized formulation over a 12-hour period. The benefits of the ionic gelation method include its straightforward operation, mild reaction conditions, and lack of need for organic solvents [30]. As a result, it has been developing really quickly lately. Many scientists are working on chitosan and its by-product nanoparticles made by the ion gelation process, which is useful for packaging macromolecules of biological origin, delivering the desired medication to the body, and releasing it gradually under control [35]. One of the upcoming developments in this field’s advancement is composite technology, which avoids the drawbacks of a particular dosage form and combines the advantages of many forms with the help of nanoparticles and other techniques [32,33].

Solvent Evaporation Method

To form the emulsion, a solution of chitosan is first incorporated into the aqueous phase. Nanospheres are then formed when the polymer solvent evaporates and precipitates. Following the addition of ethanol and deoxyribonucleic acid-tris buffer, the mix was agitated for 30 minutes using a magnetic stirrer. Nanoparticles are produced by removing the solvent at low pressure. In the end, the solvent is eliminated to create nanoparticles [36]. By letting the solvent evaporate, Novaes et al. [37] were able to generate chitosan and collagen blends with silver nanoparticles. Medical applications are compatible with the porous and interconnected architectures of the nanoparticles [38]. Additionally, this process is also used to make poly-lactic-co-glycolic acid (PLGA) nanoparticles. Subsequent research revealed that the antioxidant activity of these nanoparticles is 80%, and curcumin encapsulates 82-89% of its surface area when combined with various capping agents, including chitosan, glucan, and emulsifier. The curcumin-coated PLGA nanoparticle’s in vitro anticancer activity indicates that they are more successful at stopping cell proliferation [39]. However, the making of microspheres by decreasing pressure increases complexity and may have an impact on the active pharmaceutical component in order to increase the encapsulation rate by reducing the time spent preparing and quickening the microsphere solidification [40].

Microemulsion Method

Surfactant-rich membranes divide reverse micelles, which are liquid mixes of oil, surfactants, and water that are divided into water’s small-scale areas because they are thermodynamically more stable [30,41]. Amphiphilic molecules, known as surfactants, spontaneously assemble into spherical or ellipsoidal aggregates in organic solvents like water. Reverse micelles arise in high concentrations of solvents having organic contents, whereas typical micelles are present in H2O with low concentrations of solvents of organic origin [42].

Self-Assembling Method

A number of nanoparticles of chitosan have been used in medical settings. Nevertheless, the great majority of these conventional nanoparticles require either the use of hazardous compounds or a laborious manufacturing procedure involving high pressure, temperature, and prolonged operation [43]. The development of the self-assembly approach solved the aforementioned issues. In particular, phospholipids are negatively charged lipid mixes, while chitosan is a naturally occurring, positively charged alkaline polysaccharide [40]. Because of the electrostatic interactions, phospholipids and chitosan self-assemble to produce nanoparticles [44]. No organic solvents or cross-linking agents were used during the entire procedure, which also protected the loaded bioactive chemicals from harmful damage and handling mishaps. It is anticipated that self-assembled CNP will be extensively employed in clinical settings due to their ease of preparation, the feasibility of mass production, and high encapsulation efficiency [45]. The use of endodontic therapy is mentioned below.

Root Canal Treatment

The most prevalent type of bacteria in teeth that have had endodontic therapy is *Enterococcus faecalis*. The degree to which the bacterial burden is removed determines the outcome of a root canal. Because of its physiochemical and antimicrobial characteristics, chitosan can be used extensively in endodontic operations [46]. In addition to having a broad range of antibacterial qualities, chitosan is also biocompatible, biodegradable, and has a high chelating capacity in acidic environments. A study looked at the antimicrobial properties of the chitosan-citrate solution, which made it possible to remove the smear layer. After five minutes, the chitosan-citrate solution’s antibacterial activity and smear layer removal were both achieved, with the smear layer removal being significantly greater than the 10% concentration of citric acid. This study demonstrated that the antibacterial activity and smear layer removal properties of chitosan-citrate solution make it a potential root canal irrigant [26,43]. Common irritants include ethylenediaminetetraacetic acid (EDTA) and NaOCl. In a study, the antimicrobial activity and dentin smear layer removal capabilities of CNP were compared to those of NaOCl and EDTA. The smear layer and inorganic components from the dentin can be successfully removed by irrigation using NaOCl, according to the results. Additionally, CNP had a chelating action akin to EDTA and considerably outperformed NaOCl and EDTA in resisting the formation of biofilms [47]. Chitosan has the added advantage of being able to remineralize dentin that has become demineralized, as opposed to EDTA, which has the potential to cause dentin demineralization. CNP’s capacity to eliminate the smear layer on the dentin and prevent germs from recolonizing makes them a viable substitute for EDTA or a final irrigant in root canal therapy. Furthermore, because of its advantageous antibacterial effect, calcium hydroxide has long been the material of choice for intracanal dressings [21,26]. Additionally, chitosan can maintain calcium hydroxide’s release of calcium ions from this material. Nevertheless, calcium hydroxide loses its ability to release calcium ions and maintain an alkaline pH for longer than seven days. Consequently, when calcium hydroxide was mixed with various media, including propylene glycol, distilled water, chitosan, and gutta percha points with calcium hydroxide, a study assessed the release of calcium ions and measured the environmental change of pH [48]. In comparison to the other formula mediums, for up to 30 days, the formulation of chitosan showed the longest-lasting dispersion of ions of calcium while keeping a high alkaline pH. Thus, these investigations came to the conclusion that chitosan may be a viable calcium hydroxide delivery system for long-term dressings that maintain calcium release throughout root canal therapy. In endodontic operations, zinc-oxide eugenol (ZOE) with added chitosan can be used as a sealer or as a temporary restorative substance [47,49]. Reinfection brought on by coronal leakage is one of the frequent causes of root canal treatment failures. ZOE is frequently applied as a coronal seal to stop the spread of infection. Eugenol released by ZOE has an antimicrobial effect; however, this effect wears off with time. Chitosan is added to ZOE to investigate its antimicrobial properties against *E. faecalis*, *Streptococcus mutans*, and *Staphylococcus epidermidis.* Eugenol from ZOE is leached gradually, leading to an increase in its content, which is not advised due to its impacts on osteoblastic cells in humans, which are generally cytotoxic. The study’s findings indicate that *S. mutans *is the most eugenol-susceptible bacteria [47]. The compressive strength was decreased by adding chitosan to ZOE; however, ISO 3017’s standards for compressive strength were still met. This study provided evidence that adding chitosan to ZOE can enhance and maintain its antibacterial properties. Improving the antimicrobial capabilities of sealers can help make endodontic therapy more successful. In a study, sections of roots packed with gutta-percha and sealed with ZOE that contains CNP were investigated to see if biofilm growth occurs within the sealer-dentin interfaces [31,47]. The findings demonstrated that the biofilm development of *E. faecalis *within the sealer-dentin was prevented by adding CNP to the ZOE sealer. In canals treated with phosphorylated chitosan, the hampering impact on biofilm formation is retained; in chitosan-treated canals coupled with rose bengal and photodynamic irradiation, it is maintained to a moderate extent. According to this study, chitosan can be added to the ZOE sealer to prevent the formation of biofilm at the sealer-dentin contact [23]. Furthermore, real-seal self-etching primer can also contain chitosan to get rid of bacteria in root canals and stop subsequent infections. A study looked at the binding strength between radicular dentin and a self-etching primer enhanced with chitosan in the real-seal system, as well as the primer’s antibacterial activity. In comparison to utilizing the unmodified primer, the results showed strong antibacterial benefits against *E. faecalis *using a real-seal self-etching primer with chitosan solution without affecting bond strength [20]. Without compromising the binding strength to radicular dentin, this study showed that the real-seal system’s modified self-etching primer containing chitosan is a viable antimicrobial primer [50].

Dental Pulp Regeneration

Regenerative endodontic therapy focuses on the regeneration of pulp connective tissue, radicular dentin, vascularization, and innervation as opposed to standard endodontic treatment, which removes all pulp tissues [16]. The primary determinant of regenerative endodontic treatment is scaffolds, which are necessary to transfer active molecules and cells inside the root canal. When utilized as a scaffold, the cellularized hydrogel of fibrin with chitosan can inhibit the growth of endodontic germs and promote the development of dental pulp [20,21]. In addition to showing comparable vitality of the dental pulp, fibroblast-like pattern, and growth of collagen as normal dental tissue pulp, fibrin-chitosan hydrogel also showed strong antibacterial activity in the fibrin network. Without influencing dental pulp cell growth, form, or viability within the collagen matrix, chitosan incorporation in the hydrogel of fibrin can aid in the promotion of pulp tissue formation [11]. Due to its capacity to cause mineralization, studies have indicated that scaffolds based on chitosan are advantageous for pulp regeneration and dentin development. The presence of tricalcium phosphate in chitosan-based scaffolds stimulates the production of dentin by human periodontal ligament cells (HPLCs) and elevated mineralization expression indicators such as osteopontin along with alkaline phosphatase [11,50]. Additionally, dental pulp stem cell migration, proliferation, and odontoblastic differentiation are encouraged by scaffolds of chitosan. According to this study, chitosan-based scaffolds play a major role in the regeneration of dental pulp. An additional investigation assessed the impact of tailored chitosan or carbon dot hydrogel on the growth of stem cells from dental pulp [16,20]. The outcomes demonstrated that, in contrast to calcium hydroxide paste, hydrogels of chitosan displayed a significant degree of cellular growth. This study found that in regenerative endodontic operations, carbon dot and chitosan hydrogel can be utilized as substitutions for triple antibiotic paste or calcium hydroxide [51].

## Conclusions

Deacetylated polysaccharide chitosan has been the subject of much research due to its special properties and potential uses in dentistry, especially in endodontic treatment cases. The most significant attributes of chitosan biomaterials for endodontic therapies are their hydrophilic properties, bioactivity, biodegradable action, biocompatible nature, and antimicrobial qualities. Pulp capping agents, root canal sealers containing chitosan, and its derivatives have been used over the last few years. Chitosan biomaterials have also been investigated in recent times for use as scaffolds, hemostatic dressings, dental membranes, and carriers for medication in endodontics. Given the effectiveness and results of endodontic treatments, the growing usage of chitosan in numerous forms in the field of endodontics is being encouraged. However, further research is required to fully understand the potential and undiscovered properties of chitosan biomaterials, which could be used in regular, complex, as well as re-endodontic treatment cases.
